# 125I inhibits the progression of cervical cancer by upregulating the HSF1/PU.1/SYK signaling pathway and consequently enhancing the apoptotic response mediated by ROS/USP7/P53

**DOI:** 10.1038/s41598-025-99214-2

**Published:** 2025-05-21

**Authors:** Xiaomei Fan, Xiaoliang Liang, Yunfeng Guo, Ge Jin, Dong Ming, Xingwei An

**Affiliations:** 1 Department of Biomedical Engineering, Tianjin University, Tianjin, 300354 China; 2 Department of Gynecological Oncology, The Fourth Hospital of Hebei Medical University, Shijaizhuang, China

**Keywords:** Cervical cancer, 125I, HSF1/PU.1/SYK signaling pathway, ROS/USP7/P53 signaling pathway, Cell biology, Molecular biology

## Abstract

125I (iodine-125) is a radioactive isotope commonly used in the treatment of cervical cancer, especially in brachytherapy. This study investigates the molecular mechanism by which 125I radiotherapy inhibits the progression of cervical cancer. C33A cervical cancer cells were subjected to irradiation with 125I. Subsequently, lentiviral infection was utilized to overexpress and knock down HSF1, as well as to overexpress PU.1. The efficiency of gene knockdown was evaluated via qPCR experiments, followed by treatment with the SYK inhibitor R406. Immunofluorescence staining was employed to determine the relative fluorescence intensity of HSF1 and P53. The relative protein expression levels of HSF1, PU.1, SYK, Dectin-1, and P53 were assessed using flow cytometry to examine apoptosis in C33A cells. Cell viability and proliferative capacity were measured using CCK-8 assays and colony formation assays. The progression of cervical cancer was evaluated through subcutaneous xenograft tumor experiments in nude mice. Transwell and scratch assays were conducted to assess the invasive and migratory capabilities of C33A cells. 125I radiotherapy can augment the expression of HSF1, PU.1, SYK, Dectin-1, β3-integrin, P22, P47, P53, gp91, and p-USP7 in C33A cells. In subcutaneous xenograft tumor experiments conducted in nude mice, 125I radiotherapy also facilitates apoptosis in C33A cells, curtails their viability, proliferative potential, and invasive and migratory capacities, thereby mitigating the progression of cervical cancer. However, specific knockdown of HSF1 and the administration of the SYK inhibitor R406 can reverse the potentiating effect of 125I radiotherapy on the ROS/USP7/P53 pathway, consequently promoting the progression of cervical cancer; specific overexpression of PU.1 can abrogate the inhibitory impact of HSF1 knockdown on the ROS/USP7/P53 pathway, thereby suppressing the progression of cervical cancer. 125I inhibits the progression of cervical cancer by upregulating the HSF1/PU.1/SYK signaling pathway and consequently enhancing the apoptotic response mediated by ROS/USP7/P53.

## Introduction

Cervical cancer, as one of the malignant diseases threatening the life and health of women worldwide, ranks fourth among the common malignancies in women after breast cancer, colorectal cancer, and lung cancer. Long-term and persistent HPV infection is the main cause of cervical cancer. Due to the fact that most early cervical cancers have no obvious symptoms, most patients have already been diagnosed with locally advanced cervical cancer, so early cervical cancer screening and the use of HPV vaccines are particularly important. For patients who already have cervical cancer, the therapeutic effect remains the focus of attention for clinicians. The main treatment methods for cervical cancer patients will vary according to the different FIGO (International Federation of Gynecology and Obstetrics) stages, and radiotherapy is the main treatment modality for most patients with cervical cancer at different stages.^1–3^ While radiotherapy is highly effective, it also encompasses certain limitations. Firstly, the non-specific nature of radiotherapy can result in damage to both tumor cells and surrounding normal tissues and organs, leading to a spectrum of side effects, including radiation-induced enteritis, cystitis, and vaginal stenosis, which can substantially impair the patient’s quality of life. Secondly, cervical cancer cells exhibit a degree of resistance to radiotherapy. Some patients may not achieve complete tumor regression following standard radiation doses, and there is even the potential for recurrence or metastasis. This not only restricts the efficacy of radiotherapy but also escalates the complexity and challenge of treatment. Moreover, the long-term consequences of radiotherapy remain uncertain. Long-term follow-up studies have revealed that some patients may develop late radiation-induced injuries, such as second primary malignancies and radiation-induced cardiovascular disease, years after the completion of treatment. Although radiotherapy for cervical cancer has made great progress, the mechanism of action of radiotherapy for cervical cancer is still unclear.

In the past 20 years, with the successful development of the new radionuclide 125I particles, which is characterized by sealing, low energy, safety, and easy protection, along with the development of imaging technologies such as color ultrasound, CT, and nuclear magnetic resonance, and the emergence of computerized three-dimensional treatment planning systems (TPS), 125I radioactive particle interstitial implantation for the treatment of tumors has shown strong vitality and broad development prospects due to its efficient radiophysical dose distribution, radiobiological characteristics, reliable clinical efficacy, and little damage to surrounding normal tissues and organs, becoming another popular internal radiotherapy technique for tumor treatment.^4,5^ The physical half-life of 125I is 59.6 days, and it spontaneously decays into X-rays with an energy of 27.4–31.5 keV and γ-rays with an energy of 35.5 keV through electron capture. The interstitial implantation of 125I radioactive particles is to implant 125I radioactive particles into the tumor tissue or the tissue where tumor cells have metastasized, and kill the tumor cells by the radiation released from the decay of 125I radioactive particles. The implantation methods can be divided into direct implantation during surgery or puncture implantation under the guidance of imaging techniques such as B-ultrasound, CT, and MR. This method can make the radiation dose at the irradiated site reach the highest, while the radiation dose received by the surrounding area is small, which can improve the killing power of tumor cells. The γ-rays emitted by 125I radioactive particles have very weak penetrability, and a lead plate with a thickness of 1 mm can block more than 99% of the γ-rays. For medical personnel, the radiation only comes from external exposure, which greatly improves the safety factor of the operator during the implantation of radioactive 125I particles, and the patients undergoing this treatment are also safe, so 125I radioactive particles are a very suitable radioactive isotope for permanent interstitial implantation.^6–8^

Radiotherapy uses high-energy radiation to directly act on the tumor tissue, causing ionization of water molecules in the tumor cells and generating a large amount of reactive oxygen species (ROS) and free radicals. These ROS and free radicals cause severe oxidative stress in tumor cells, leading to imbalance in the intracellular redox balance. ROS and free radicals can directly react with DNA molecules, causing severe damage such as DNA double-strand breaks, single-strand breaks, and base modifications. If these DNA damages cannot be repaired in time, they will lead to cell cycle arrest, mutations, or cell apoptosis. ROS can also oxidize proteins and lipids in the cell, causing protein denaturation, enzyme inactivation, and membrane damage. These damages will further disrupt cell function and promote cell death.^9–11^ Oxidative stress also activates a series of cellular stress responses and signaling pathways, such as p53, NF-κB, and MAPK. These signaling pathways can initiate cell death mechanisms such as apoptosis (programmed cell death) or necrosis.^12,13^ Under oxidative stress conditions, BRD4 (a bromodomain and extraterminal domain protein) is activated. BRD4 is an important epigenetic regulator that can regulate gene expression by binding to acetylated histones. The activation of BRD4 can further activate the endoplasmic reticulum stress response (ERS). Some studies have found that endoplasmic reticulum stress can promote the expression of HSF1. As one of the main transcriptional regulators of the heat shock response (HSR), heat shock factor 1 (HSF1) plays an important role when cells and organs are exposed to heat, ischemia, hypoxia, inflammation, and other adverse stress factors.^14–17^ This study endeavors to elucidate the cytotoxic effects of 125I particle radiotherapy on cervical cancer cells and the underlying mechanisms, particularly through the upregulation of the HSF1/PU.1/SYK signaling pathway, which subsequently enhances the ROS/USP7/p53-mediated apoptotic response and inhibits cervical cancer progression. Through this investigation, we aspire to establish new theoretical foundations and practical guidance for cervical cancer radiotherapy, thereby improving therapeutic efficacy, mitigating side effects, and enhancing patient outcomes and quality of life.

## Methods

### Bioinformatics analysis

Download the cervical cancer data set GSE56363 from the Gene Expression Omnibus (GEO) database. GSE56363 includes 12 patient samples with a complete response after 6 months of chemotherapy and 9 patient samples with a non-complete response after 6 months of chemotherapy. Download and filter oxidative stress-related genes with a Relevance score > 7 in the GeneCards database, and a total of 1482 related genes were extracted. In terms of data preprocessing, RNA sequencing data was first standardized and transformed using log2, and the chip data in the GEO data set was background corrected, normalized, and multi-probe averaged using RMA (Robust Multi-array Average). The processed data was batch effect corrected using the Combat method. The screening program for differential molecules was performed through the normalized data, and the differential expression analysis was performed using the online analysis tool GEO2R. The screening criteria were: genes with |log2 Fold Change|> 1 and p-value < 0.05 were considered differentially expressed genes. The p-value was corrected for multiple hypothesis tests using the Benjamini–Hochberg method. Gene Ontology (GO) and Kyoto Encyclopedia of Genes and Genomes (KEGG) pathway enrichment analyses were performed using the ClusterProfiler package. GO analysis mainly includes Cellular Component (CC), Molecular Function (MF), and Biological Process (BP); while KEGG pathway analysis is used to reveal key biological pathways. Analyze the correlation of differential genes by drawing a correlation scatter plot.^18–21^

### Cell culture

Cervical cancer cells C33A were purchased from Wuhan Proscience Biotechnology Co., Ltd. C33A cells were maintained in DMEM medium containing 10% FBS (Gibco; Thermo Fisher Scientific) and 1% penicillin/streptomycin (Gibco; Thermo Fisher Scientific), with a CO_2_ content of 5% and an atmospheric temperature of 37 ℃.

### Cell treatment

C33A and HeLa cells were separately seeded into 6-well plates. Once the cells had adhered, the NC group, serving as the control, received no additional treatment. Plasmids for packaging HSF1 overexpression lentivirus, HSF1 shRNA lentivirus, and PU.1 overexpression lentivirus, including the psPAX2 plasmid, were individually transfected into HEK293T cells using Lipofectamine 3000. The supernatant containing viral particles was collected 48 h post-transfection. The viral titer was subsequently amplified through ultracentrifugation. The lentiviral supernatants were then added to C33A and HeLa cells according to the designated groups: the HSF1-OE group received the HSF1 overexpression lentiviral supernatant; the HSF1-KD group received the HSF1 shRNA lentiviral supernatant; and the HSF1-KD + PU.1-OE group received the PU.1 overexpression lentiviral supernatant. After 24 h of infection, the medium was replaced with fresh medium to mitigate viral cytotoxicity. At 48 h post-infection, the appropriate antibiotics were introduced to select for successfully infected cells, ensuring the survival of only the infected cells. Then, the cells were treated with 125I particle radiotherapy.

The 125I radioactive particle (0.8 mCi, model 6711) radiation model consisted of a lower irradiation plane and an upper treatment plane (the bottom of a 35-mm polystyrene culture dish). The height between the irradiation plane and the treatment plane was 6 mm. On the irradiation plane, 8 seeds with the same activity were evenly distributed on the circumference with a diameter of 35 mm, and the ninth seed was confined to the center. The initial dose rate of the treatment plane was 2.7 cGy/h, providing a cumulative radiation dose of 6 Gy. Therefore, the C33A cells were divided into a control group and four experimental groups for this experiment. The control group received no additional treatment, while the experimental groups were as follows: 125I group, NC-HSF1-OE + 125I group, HSF1-OE + 125I group, NC-HSF1-KD + 125I group, HSF1-KD + 125I group, HSF1-KD + 125I + PU.1-OE group, and HSF1-KD + 125I + PU.1-OE + R406 group (R406 is a SYK inhibitor).

### Q-PCR was used to validate the knockdown efficiency

Total RNA was extracted from C33A and HeLa cells following culturing using Trizol reagent. To quantify the expression of HSF1 and PU.1 in H446 cells, cDNA was synthesized using the PrimeScript™ RT Kit. qPCR was conducted using SYBR Green qPCR Master Mix and the ABI StepOne system, with an amplification program comprising enzyme activation at 95 °C for 30 s, followed by denaturation at 95 °C for 15 s, annealing and extension at 60 °C for 30 s, and a final extension at 72 °C for 30 s. Post-reaction, the amplification curves of HSF1 and PU.1 were verified to ensure primer specificity. The primers used are as follows:

**Table:** 

Gene	Forward primer sequence (5' → 3')	Reverse primer sequence (5'–3')
HSF1	TGAGAATGAGGCTCTGTGG	ACTGAATGAGCTTGTTGACG
PU.1	GTGCCCTATGACACGGATCTA	AGTCCCAGTAATGGTCGCTAT

### Cell slide preparation

Took C33A cells in the logarithmic growth phase, made a single-cell suspension after digestion, adjusted the cell concentration to 5 × 104 cells/mL, inoculated 2–3 drops of the cell suspension on the autoclaved glass slide, put the glass slide in the autoclaved wet box, and continued to incubate at 37 ℃ and 5% CO_2_ for 24 h. After that, a large number of cells grew on the glass slide to form a cell slide.

### Immunofluorescence staining experiment

After washing the cells three times with PBS, 4% paraformaldehyde was added and fixed at room temperature for 15 min. 0.3% Triton X-100 was added and permeabilized at room temperature for 10 min, then 5% goat serum was added for blocking for 1 h. Then the primary antibody diluted with 5% goat serum was added, and incubated at 4 ℃ overnight. Washed three times with PBS, the secondary antibody diluted with 5% goat serum was added, and incubated at room temperature for 1 h. Finally, DAPI was added and incubated for 10 min. After washing three times with PBS, it was sealed and pictures were taken.

### Flow cytometry

After digesting the podocytes with trypsin–EDTA digestion solution (0.25%), centrifuged at room temperature (1000 r/min, 5 min), the supernatant was discarded, and washed with 1xPBS. Approximately 1 × 106 C33A cells were collected in each group, 500 μl binding buffer was added, blown and mixed evenly, 5 μl PI and Annexin V-FITC were added respectively, and let stand in the dark for 10 min. Apoptosis of C33A cells was detected by flow cytometry.

### CCK-8 assay

Took C33A cells in good growth condition, digested and counted, and inoculated 1 × 104 cells/well in 96-well plates. After overnight culture, 10 μL of CCK8 solution was added to each well, and a multifunctional microplate reader was used to detect the absorbance value at a wavelength of 450 nm after incubation for 24 h, 48 h, and 72 h.

### Clone formation assay

Took C33A cells in good growth condition, digested and counted, and seeded them in a 6-well plate, about 1000 cells per well. After the cells adhered to the wall and grew for 3 days, the medium was changed, and the culture was continued for 15 days. After visible clone formation with the naked eye, PBS was washed, fixed with paraformaldehyde for 30 min, added ready-to-use crystal violet solution for staining overnight, washed with PBS, and pictures were taken and relevant data of each well was saved under a microscope.

### Western blot experiment

Took C33A cells in good growth condition, digested and counted, and planted 3 × 105 cells per well in a 6-well plate, and incubated overnight. On the next day, after 48 h of treatment with the drug solution prepared in the complete medium, the 6-well plate was removed from the incubator, washed with 1xPBS, and an appropriate amount of cell lysis buffer was added according to the amount of cells. After lysing for 30 min, the cells in each group were scraped with a cell scraper, centrifuged and quantified, and each group of protein samples (30 μg) was separated with 10% SDS-PAGE, 80 V for 30 min for the concentrated gel, and 110 V for 50 min for the separation gel. After that, the protein samples were transferred to the PVDF membrane and the membrane was transferred. After the membrane transfer was completed, it was sealed with 5% milk powder for 2 h, the primary antibody was incubated overnight, the secondary antibody was incubated for 1 h on the second day, washed with TBST, and exposed and the grayscale value was detected using imageJ.

### Subcutaneous xenograft tumor experiment in nude mice

BALB/c male nude mice were purchased from Henan SKBS Biotechnology Co., Ltd. 4 weeks old, with a body weight of (18 ± 3) g, the nude mice were housed in an environment with a temperature (24 ± 2) ℃ and a relative humidity of 50–60%, with free diet and 12-h light–dark alternation, and were adaptively fed for 1 week. A total of 30 nude mice were randomly divided into 5 groups of 6 mice each. Mice were gas anesthetized with 3% isoflurane to ensure anesthesia was maintained for 1.5%. C33A cells in the logarithmic growth phase were taken and their density was adjusted to 1 × 104 L-1, and 200 μL of single-cell suspension was uniformly inoculated subcutaneously on the back of the nude mice’s neck. The tumor volume was calculated 20 days later and the tumor weight was measured. The formula for calculating tumor volume is: V = π/6 × L × W × H. Subsequently, the nude mice were observed and euthanized with carbon dioxide. All methods in this experiment conformed to the ARRIVE guidelines. All methods were performed in accordance with the relevant guidelines and regulations.

### Assessment of cell migration and invasion capacity

The 24-well plates and Transwell inserts were procured from Corning-Costar, USA. Cells were seeded and cultured in the upper chambers of the Transwells using fresh, serum-free EMEM, while the lower 24-well plates contained EMEM supplemented with 20% FBS. After 48 h, the cells in the upper chambers were washed with PBS and fixed with 4% methanol. Non-migrated cells were removed with PBS, and the migrated cells were stained with crystal violet. The number of cells was quantified using ImageJ software.

Cells were cultured in 12-well plates to achieve confluence, and a straight wound was created on the surface by gently scraping with a 200 μL pipette tip. Debris was removed with PBS, and fresh, serum-free EMEM was added. Wound images were captured at 0 and 48 h, and the gap width was analyzed using ImageJ software. The grouping of experimental mice corresponded to the grouping of cellular experiments, with six experimental mice in each group for the experiments. The migration rate was calculated using the following formula: migration rate = [(gap width at 0 h) − (gap width at 48 h)]/(gap width at 0 h) × 100.

### Statistical analysis

Used GraphPad Prism 9.0 for statistical analysis. The results were presented as mean ± standard deviation. Comparisons between different groups were performed using independent sample t-tests. A *p*-value < 0.05 was considered statistically significant.

## Results

### Bioinformatics results

**Fig. 1:**
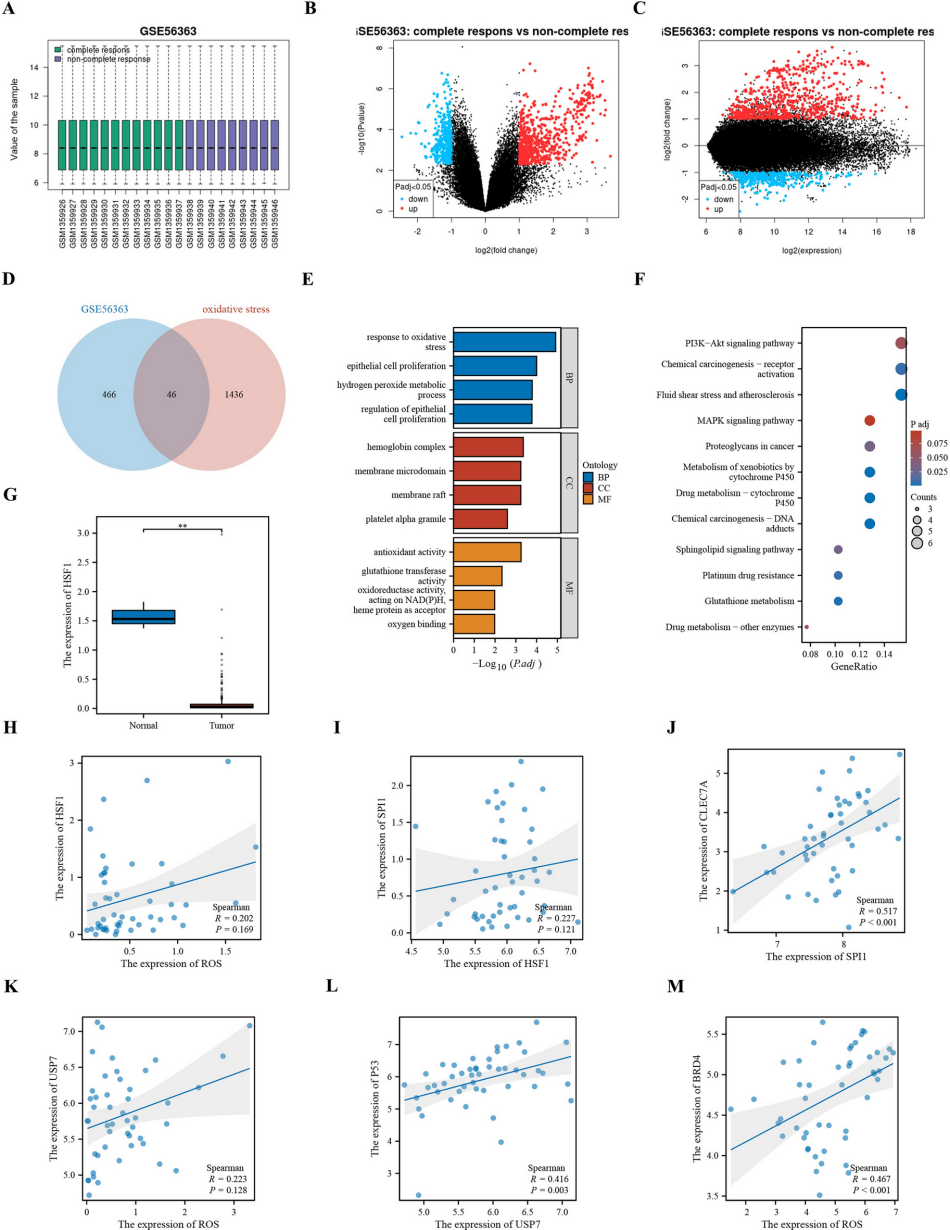
Bioinformatics analysis. (**A**) Box plot of GSE56363. (**B**) Volcano plot of GSE56363. (**C**) Mean difference plot of GSE56363. (**D**) Venn diagram of differentially expressed genes and oxidative stress-related genes in GSE56363. (**E**) Bar graph of GO enrichment analysis. (**F**) Bubble plot of KEGG enrichment analysis. (**G**) Expression of HSF1 in cervical cancer. (**H**) Correlation scatter plot between ROS and HSF1. (**I**) Correlation scatter plot between HSF1 and PU.1. (**J**) Correlation scatter plot between PU.1 and Dectin1. (**K**) Correlation scatter plot between ROS and USP7. (**L**) Correlation scatter plot between USP7 and P53. (**M**) Correlation scatter plot between ROS and BRD4.

In the data set GSE56363, 334 significantly upregulated genes and 178 significantly downregulated genes were discovered. Through the Venn diagram, 46 cross genes were found, of which 39 were significantly upregulated genes, and we hypothesized that chemotherapy leads to oxidative stress. GO and KEGG analyses of differential genes were performed using ClusterProfiler, and it was found that DEGs were significantly enriched in multiple biological pathways. GO analysis showed that DEGs were enriched in hemoglobin complex, DNA repair complex, and membrane raft in the Cellular Component; antioxidant activity, glutathione transferase activity, and oxygen binding in the Molecular Function; and response to oxidative stress, epithelial cell proliferation, and hydrogen peroxide metabolic process in the Biological Process. KEGG pathway analysis revealed that DEGs were significantly enriched in the PI3K-Akt signaling pathway, Chemical carcinogenesis—DNA adducts, and MAPK signaling pathway, etc. Through correlation analysis, it was found that HSF1 is lowly expressed in cervical cancer, ROS can promote the expression of HSF1, HSF1 promotes the expression of PU.1 (SPI1), PU.1 (SPI1) promotes the expression of Dectin1 (CLEC7A), ROS promotes the expression of USP7, USP7 promotes the expression of P53, and ROS promotes the expression of BRD4 (Fig. 1).

### The verification of lentivirus transduction efficiency by qPCR.

**Fig. 2:**
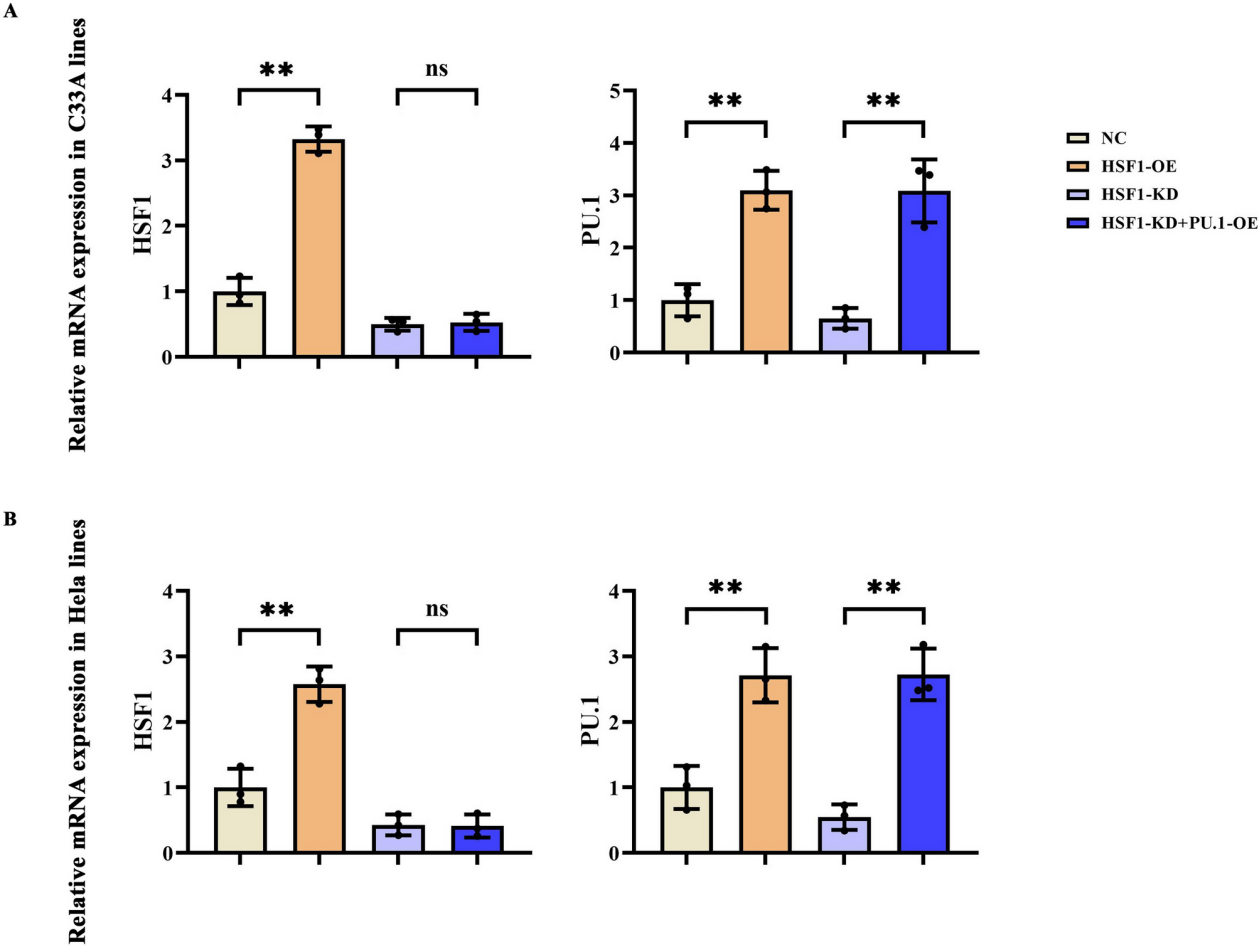
Q-PCR assay validates knockout efficiency. (**A**) mRNA expression levels of HSF1 and PU.1 in C33A cells. (**B**) mRNA expression levels of HSF1 and PU.1 in Hela cells. *p* < 0.05 was considered statistically significant. ^ns^*P* > 0.05; **P < 0.01.

Through qPCR experiments, it was determined that the mRNA levels of HSF1 and PU.1 in both C33A and HeLa cells in the HSF1-OE group were significantly elevated compared to the NC group. In the HSF1-KD group, the mRNA levels of HSF1 and PU.1 in both C33A and HeLa cells were markedly reduced. In the HSF1-KD + PU.1-OE group, the mRNA level of HSF1 in both C33A and HeLa cells was significantly decreased, while the mRNA level of PU.1 was significantly increased. However, the transduction efficiency in C33A cells was slightly higher than that in HeLa cells. Moreover, C33A cells exhibit a higher proliferation capacity and relatively better genetic stability, rendering them more reliable for subsequent experiments. Therefore, C33A cells were selected for further experiments in this study (Fig. 2).

### 125I particle radiotherapy enhances the process of ROS/USP7/P53 by promoting the HSF1/PU.1/SYK signaling pathway

**Fig. 3:**
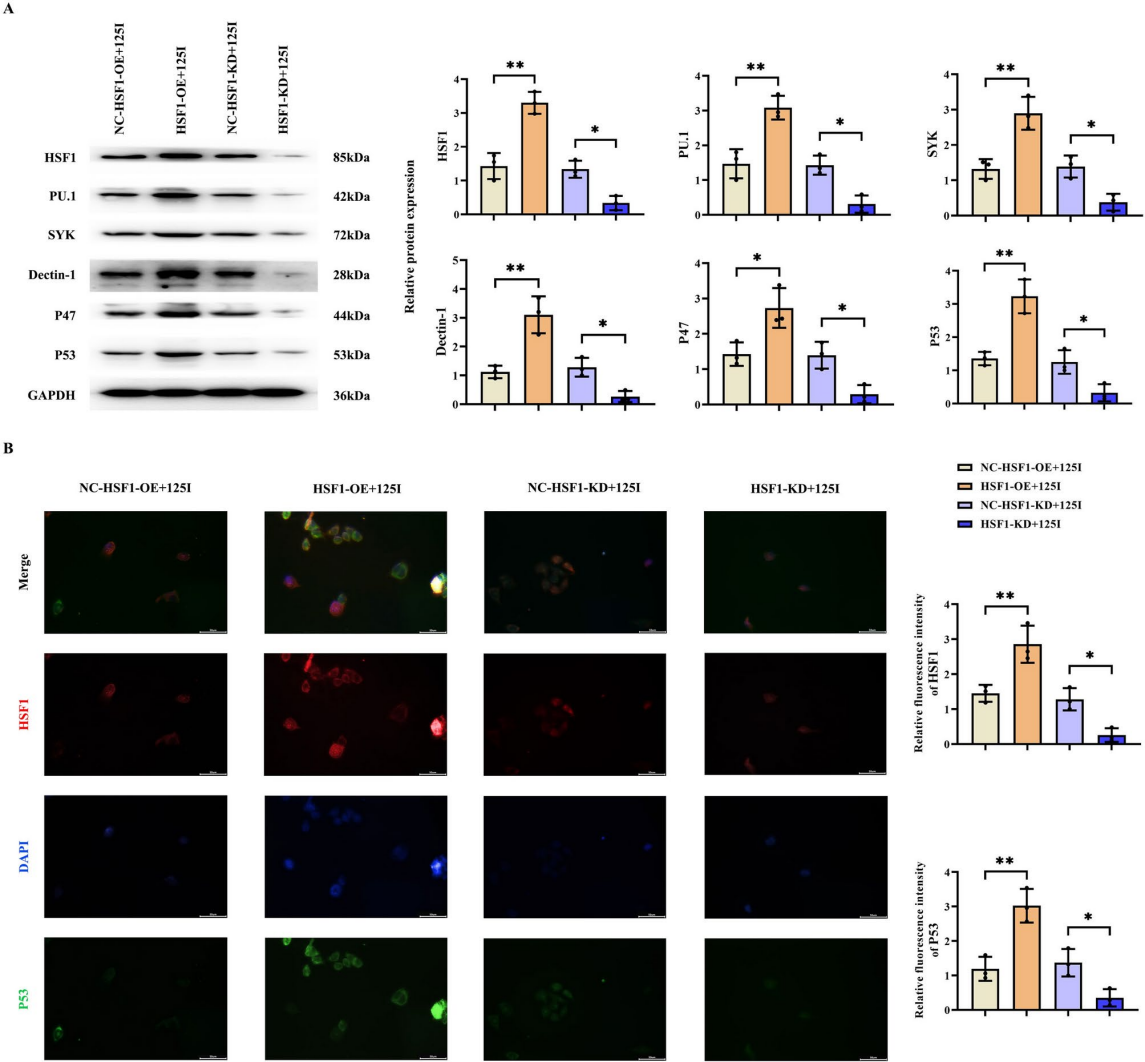
The role of HSF1 in the process of 125I particle radiotherapy-induced oxidative stress. (**A**) Protein bands and relative protein expression levels of HSF1, PU.1, SYK, Dectin-1, β3-integrin, P22, P47, and P53, with GAPDH used as a loading control. (**B**) Immunofluorescence staining results and relative fluorescence intensity statistics of HSF1 and P53. Data are presented as the mean ± standard deviation. N = 3, and *P* < 0.05 is considered statistically significant. *P < 0.05; **P < 0.01.

To elucidate the role of HSF1 in the 125I particle radiotherapy process for the treatment of cervical cancer, we silenced or overexpressed HSF1 in C33A cells and subjected them to 125I particle radiotherapy. The results of the Western blot experiment demonstrated that the relative protein expression levels of HSF1, PU.1, SYK, Dectin-1, β3-integrin, P22, P47, and P53 in the HSF1-OE + 125I group were markedly elevated compared to those in the NC-HSF1-OE + 125I group. Conversely, the relative protein expression levels of HSF1, PU.1, SYK, Dectin-1, β3-integrin, P22, P47, and P53 in the HSF1-KD + 125I group were significantly reduced relative to those in the NC-HSF1-KD + 125I group. Subsequently, we prepared C33A cell slides and employed immunofluorescence staining to measure the relative fluorescence intensities of HSF1 and P53. The results indicated that the relative fluorescence intensities of HSF1 and P53 in the HSF1-OE + 125I group were notably higher than those in the NC-HSF1-OE + 125I group (Fig. 3). In the HSF1-KD + 125I group, the relative fluorescence intensities of HSF1 and P53 were significantly lower compared to those in the NC-HSF1-KD + 125I group. Thus, it was confirmed that HSF1 plays a crucial role in the 125I particle radiotherapy-induced oxidative stress.

**Fig. 4:**
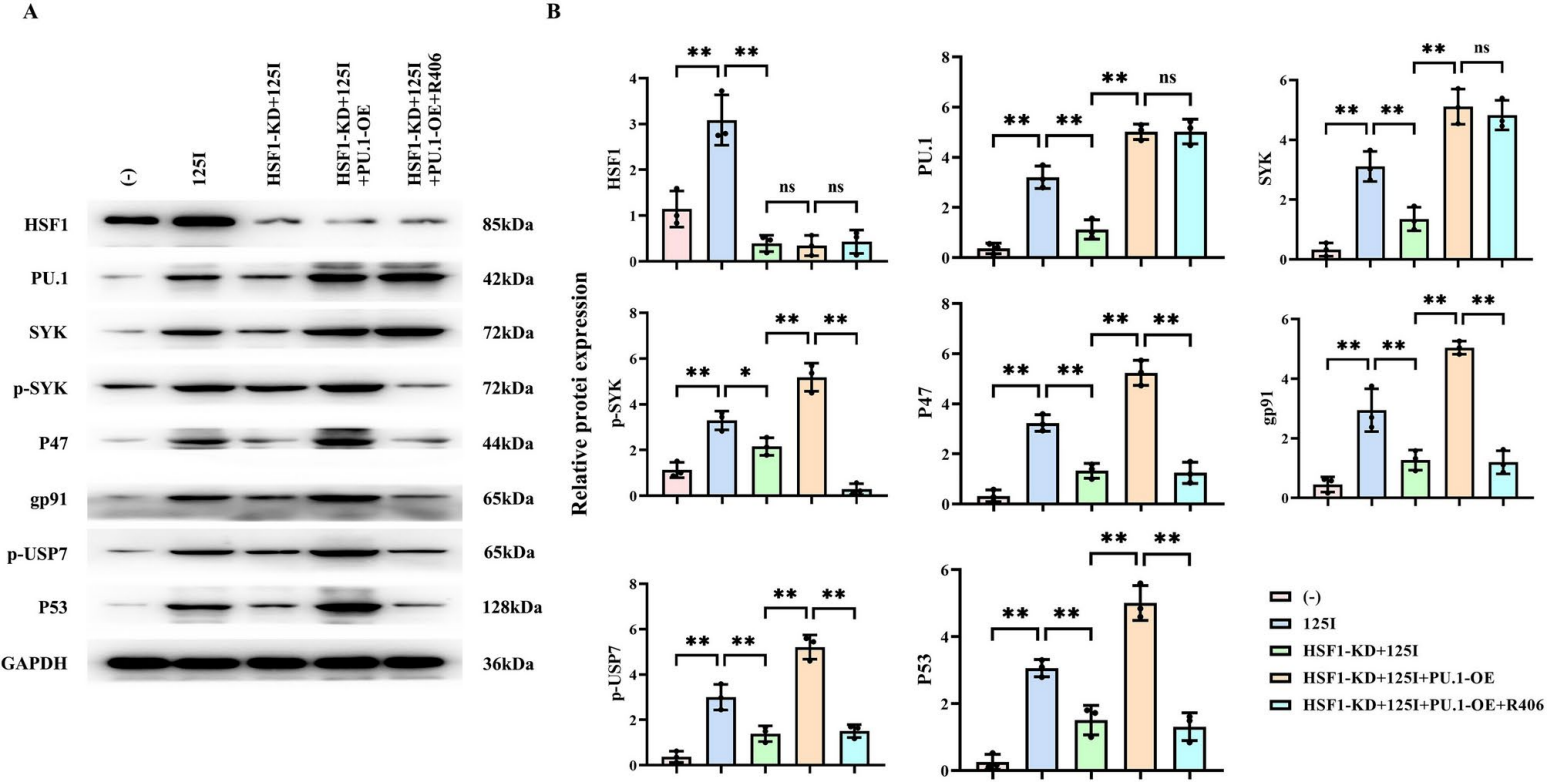
The effect of 125I particle radiotherapy on promoting the HSF1/PU.1/SYK signaling pathway and ROS/USP7/P53. (**A**) Protein bands of HSF1, PU.1, SYK, p-SYK, P47, gp91, p-USP7, and P53. (**B**) Relative protein expression levels of HSF1, PU.1, SYK, p-SYK, P47, gp91, p-USP7, and P53. GAPDH is used as a loading control, and data are presented as the mean ± standard deviation. N = 3, and *P* < 0.05 is considered statistically significant. *P < 0.05; **P < 0.01; ^ns^*P* > 0.05.

We then established a control group and a 125I group, and overexpressed PU.1 and added R406 to the C33A cells in the HSF1-KD + 125I group. The Western blot results revealed that the relative protein expression levels of HSF1, PU.1, SYK, p-SYK, P47, gp91, p-USP7, and P53 in the 125I group were significantly higher than those in the control group. In the HSF1-KD + 125I group, the relative protein expression levels of HSF1, PU.1, SYK, p-SYK, P47, gp91, p-USP7, and P53 were markedly lower compared to those in the 125I group. Moreover, compared to the HSF1-KD + 125I group, there was no significant difference in the relative protein expression level of HSF1 in the HSF1-KD + 125I + PU.1-OE group, while the relative protein expression levels of PU.1, SYK, p-SYK, P47, gp91, p-USP7, and P53 were significantly elevated. Compared with the HSF1-KD + 125I + PU.1-OE group, there was no significant difference in the relative protein expression levels of HSF1, PU.1, and SYK in the HSF1-KD + 125I + PU.1-OE + R406 group, whereas the relative protein expression levels of p-SYK, P47, gp91, p-USP7, and P53 were notably decreased (Fig. 4). In conclusion, these findings indicate that 125I particle radiotherapy enhances the expression of ROS, USP7, and P53 by activating the HSF1/PU.1/SYK signaling pathway.

### 125I particle radiotherapy enhances the apoptosis of C33A cells and inhibits the proliferation of C33A cells by promoting the HSF1/PU.1/SYK signaling pathway

Subsequently, we employed flow cytometry to investigate the mechanism by which 125I particle radiotherapy influences apoptosis in C33A cells. The results revealed that the apoptosis rate in the 125I group was markedly higher than that in the control group. The apoptosis rate in the HSF1-KD + 125I group was significantly lower compared to the 125I group. In contrast, the apoptosis rate in the HSF1-KD + 125I + PU.1-OE group was substantially increased relative to the HSF1-KD + 125I group. Compared with the HSF1-KD + 125I + PU.1-OE group, the apoptosis rate in the HSF1-KD + 125I + PU.1-OE + R406 group was notably decreased.

**Fig. 5:**
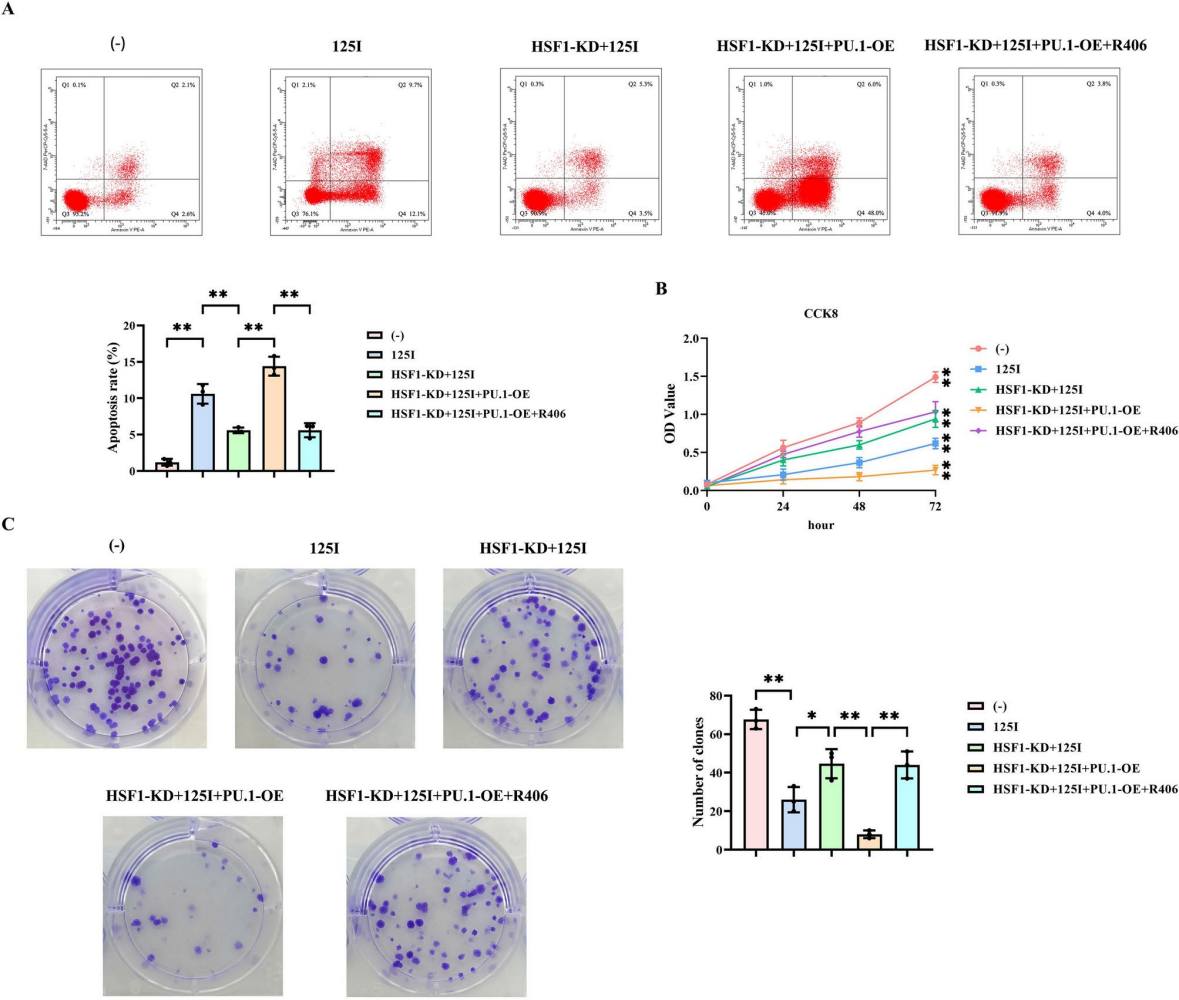
The effect of 125I particle radiotherapy on the apoptosis and proliferation of C33A cells. (**A**) Results of flow cytometry for detecting the apoptosis of C33A cells and statistics of the apoptosis rate of C33A cells. (**B**) Results of the CCK-8 assay for detecting the viability of C33A cells. (**C**) Results of the clone formation assay for detecting the proliferation ability of C33A cells and statistics of the number of clones. Data are presented as the mean ± standard deviation. N = 3, and *P* < 0.05 is considered statistically significant. *P < 0.05; **P < 0.01.

We then utilized the CCK-8 assay and the clone formation assay to evaluate the impact of 125I particle radiotherapy on the viability and proliferative capacity of C33A cells. The results of the CCK-8 assay demonstrated that at 72 h, the OD value in the 125I group was significantly lower than that in the control group. The OD value in the HSF1-KD + 125I group was markedly higher than that in the 125I group. In comparison with the HSF1-KD + 125I group, the OD value in the HSF1-KD + 125I + PU.1-OE group was substantially decreased. Compared with the HSF1-KD + 125I + PU.1-OE group, the OD value in the HSF1-KD + 125I + PU.1-OE + R406 group was significantly increased. The results of the clone formation assay showed that the number of clones in the 125I group was substantially lower than that in the control group. The number of clones in the HSF1-KD + 125I group was markedly higher compared to the 125I group. In contrast, the number of clones in the HSF1-KD + 125I + PU.1-OE group was significantly reduced relative to the HSF1-KD + 125I group. Compared with the HSF1-KD + 125I + PU.1-OE group, the number of clones in the HSF1-KD + 125I + PU.1-OE + R406 group was notably increased (Fig. 5). In conclusion, these findings confirm that 125I particle radiotherapy enhances apoptosis and suppresses the proliferation of C33A cells by activating the HSF1/PU.1/SYK signaling pathway.

### 125I particle radiotherapy inhibits the progression of cervical cancer by promoting the HSF1/PU.1/SYK signaling pathway

**Fig. 6:**
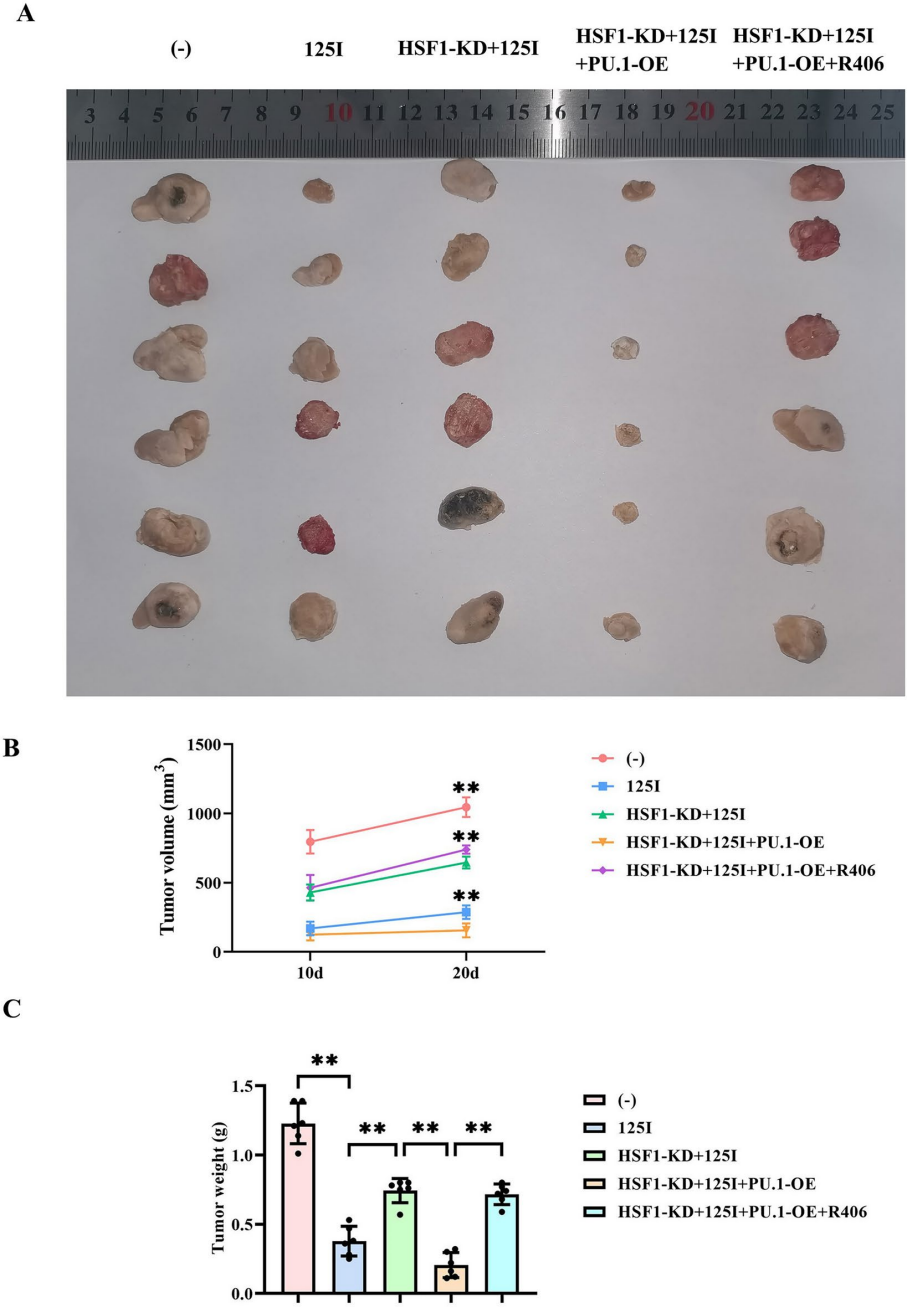
The effect of 125I particle radiotherapy on the progression of cervical cancer. (**A**) Results of the subcutaneous xenograft tumor experiment in nude mice for detecting the progression of cervical cancer. (**B**) Statistical results of the tumor volume of nude mice. (**C**) Statistical results of the tumor weight of nude mice. Data are presented as the mean ± standard deviation. N = 6, and *P* < 0.05 is considered statistically significant. **P < 0.01.

Finally, we used the subcutaneous xenograft tumor experiment in nude mice to verify that 125I particle radiotherapy inhibits the progression of cervical cancer by promoting the HSF1/PU.1/SYK signaling pathway. The results showed that both the tumor volume and weight were significantly reduced after 125I particle radiotherapy. The tumor volume and weight of C33A cells treated with 125I particle radiotherapy after specifically inhibiting HSF1 increased significantly, but were smaller than those in the control group. Continuing to specifically overexpress PU.1 under this condition, the tumor volume and weight decreased significantly, even smaller than those in the 125I group (Fig. 6). Finally, after adding R406, the tumor volume and weight increased significantly.

### 125I particle radiotherapy inhibits cervical cancer invasion and migration by promoting HSF1/PU.1/SYK signaling pathway

**Fig. 7:**
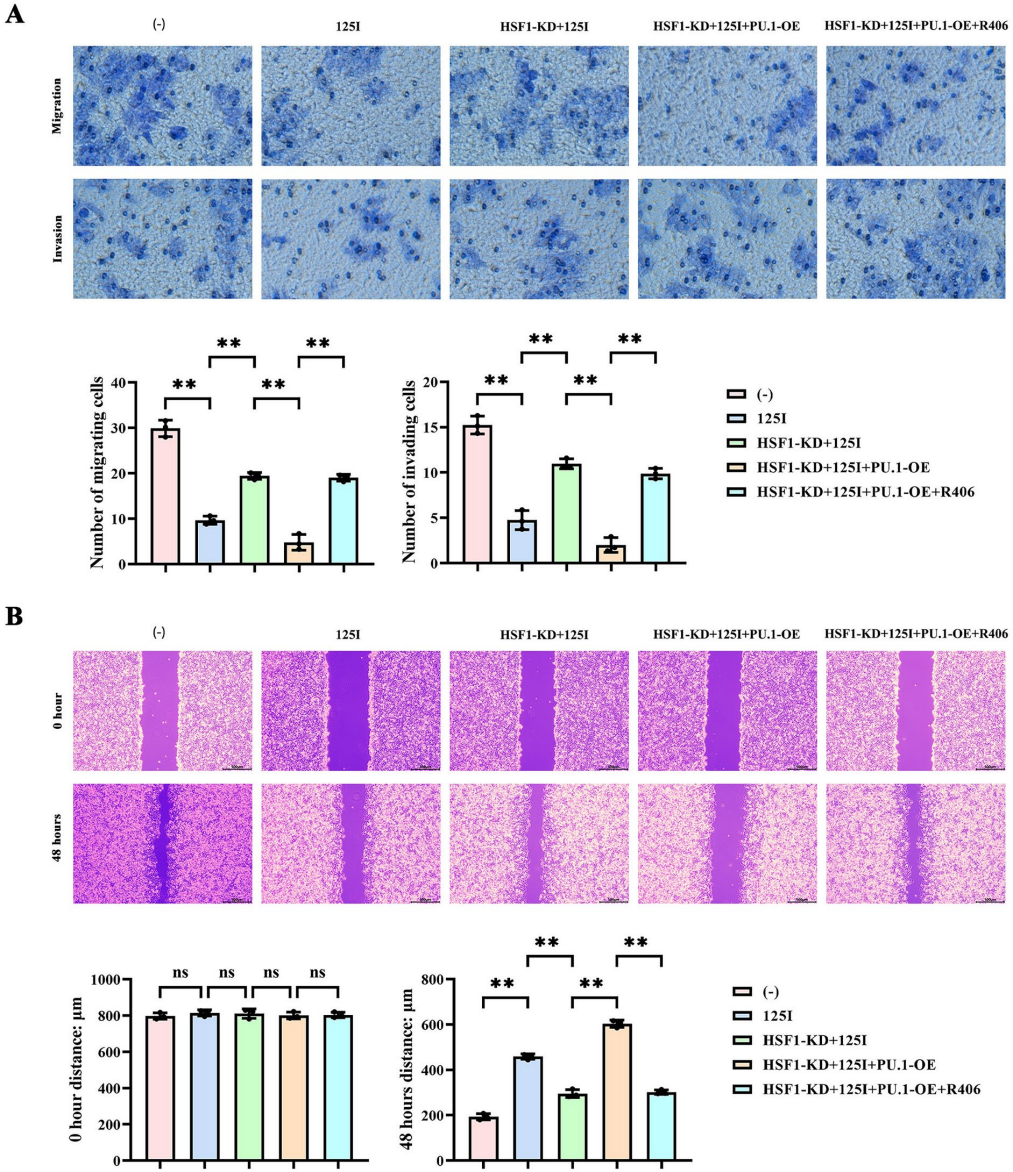
Effect of 125I particle radiotherapy on invasive migration of cervical cancer. (**A**) Transwell detection of invasion and migration ability of C33A cells. (**B**) Scratch assay to detect the migration ability of C33A cells. *p* < 0.05 was considered statistically significant. nsP > 0.05; **p < 0.01.

**Fig. 8:**
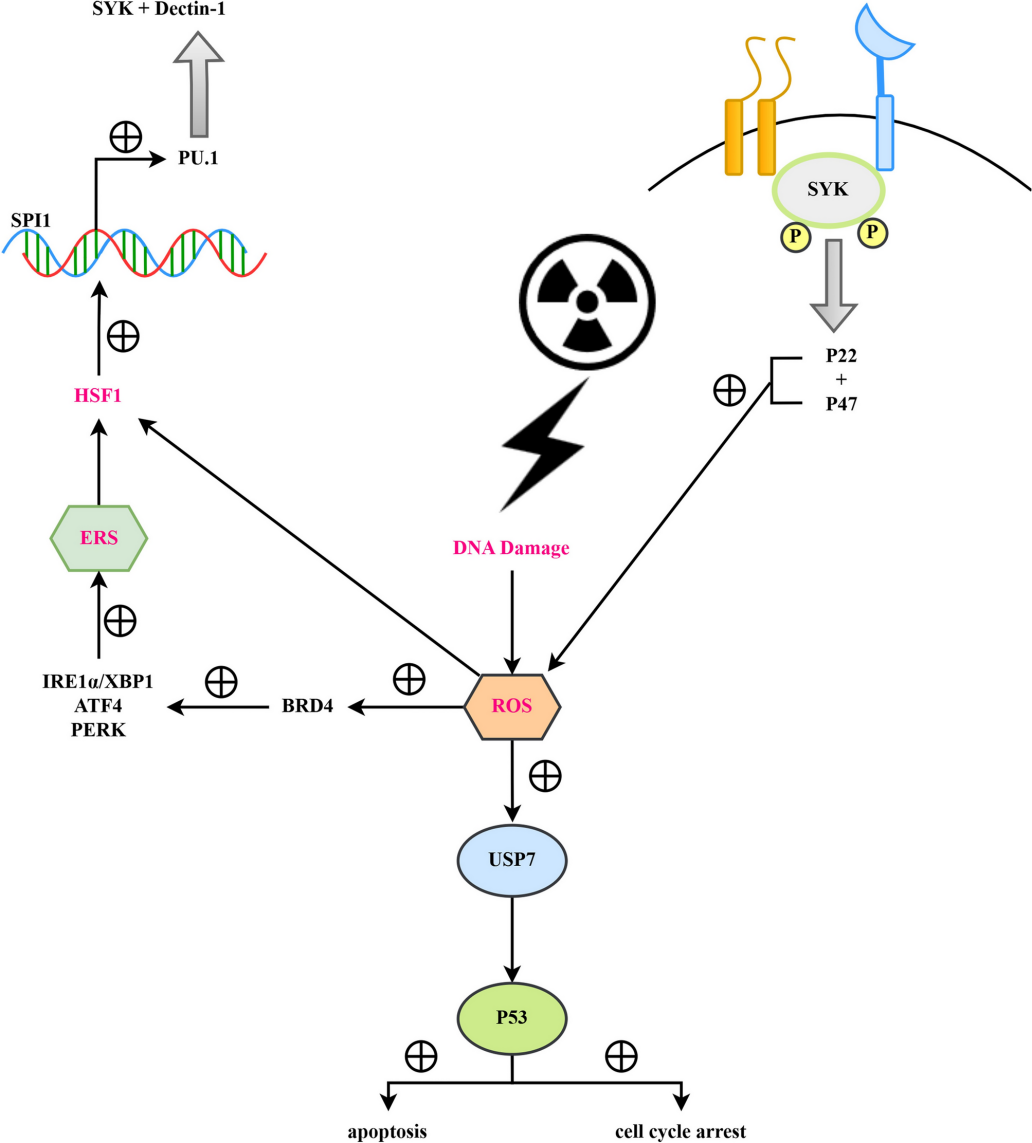
125I particles inhibit the progression of cervical cancer by up-regulating the HSF1/PU.1/SYK signaling pathway and enhancing the ROS/USP7/P53-mediated apoptotic response.

The number of invasive and migratory tumor cells in the NC group was significantly higher than in the 125I group; the number of invasive and migratory tumor cells in the HSF1-KD + 125I group was significantly higher than in the 125I group; the number of invasive and migratory tumor cells in the HSF1-KD + 125I + PU.1-OE group was significantly lower than in the HSF1-KD + 125I group; the number of invasive and migratory tumor cells in the HSF1-KD + 125I + PU.1-OE + R406 group was significantly higher than in the HSF1-KD + 125I + PU.1-OE group. The scratch width of tumor cells in the NC group was significantly narrower than in the 125I group; the scratch width of tumor cells in the HSF1-KD + 125I group was significantly narrower than in the 125I group; the scratch width of tumor cells in the HSF1-KD + 125I + PU.1-OE group was significantly wider than in the HSF1-KD + 125I group; the scratch width of tumor cells in the HSF1-KD + 125I + PU.1-OE + R406 group was significantly narrower than in the HSF1-KD + 125I + PU.1-OE group. In summary, 125I particles inhibited the invasive and migratory capabilities of cervical cancer by upregulating the HSF1/PU.1/SYK signaling pathway and enhancing the ROS/USP7/P53-mediated apoptotic response (Fig. 7). In summary, 125I particles inhibit the progression of cervical cancer by up-regulating the HSF1/PU.1/SYK signaling pathway and enhancing the ROS/USP7/P53-mediated apoptotic response (Fig. 8).

## Discussion

At present, the main treatment method for early cervical cancer is surgical treatment, and the standard treatment plan is radical hysterectomy (RH) combined with pelvic lymph node dissection (PL), and para-aortic lymph node sampling may be performed if necessary. The traditional treatment methods have their limitations: surgical trauma is relatively large, and the rate of re-radical resection is low; chemotherapy is not ideal due to local fibrous tissue hyperplasia after surgery or radiotherapy, and it is difficult for drugs to be absorbed. The side effects of radiotherapy are large, and patients are difficult to tolerate. 125I radioactive particle implantation therapy has developed rapidly in recent years, and it has been favored by clinicians due to its small trauma, definite curative effect, and few complications.^22^ However, exploring the molecular mechanism of 125I radiotherapy provides a new treatment idea and target for the radiotherapy process.

In vivo implantation of radioactive 125I particles is a treatment method for internal radiotherapy, which destroys and kills tumor tissues by continuously releasing low-energy gamma rays, thereby controlling tumor growth, and has the characteristics of less harm to patients and significant therapeutic effect. Its therapeutic mechanism may include: continuous release of gamma rays can change the expression pattern of DNA methyltransferase, thereby inhibiting tumor cell growth and inducing tumor cell apoptosis; gamma rays can generate free radicals through ionization, thereby damaging tumor cells; continuous irradiation of 125I particles can reduce tumor microvessel density and inhibit tumor vascular endothelial growth factor expression, achieving the purpose of destroying tumor microvessels and inhibiting tumor angiogenesis, thereby reducing the blood supply of tumors; gamma rays can directly destroy the DNA double strand of tumor cells, destroy the reproductive ability of tumor cells, and then control tumor growth.^23–25^

HSF1 is a major regulator of the heat shock response, which can program the stromal cells of the tumor, thereby promoting the formation of malignant tumors. HSF1 can be activated by high temperature, heavy metals, low pH value, etc., thereby causing damage to the proteins in the cells. Some studies have found that ROS can promote the activation of endoplasmic reticulum stress by activating BRD4. Endoplasmic reticulum stress is a cellular response mechanism. When unfolded or misfolded proteins accumulate in the endoplasmic reticulum, the endoplasmic reticulum stress response is activated, trying to restore the normal function of the endoplasmic reticulum. In the endoplasmic reticulum stress response, some signaling pathways, such as IRE1α, PERK, and ATF6, will be activated, initiating a series of gene expression changes. Among these changes, the heat shock response (HSR), in which HSF1 is activated as the main regulatory factor, is included.

As a transcription factor, HSF1 can cause structural and functional remodeling of the tumor stroma. As a transcription factor, HSF1 can directly bind to the promoter region or enhancer region of the PU.1 gene, enhancing the transcription and expression of PU.1.^26^ PU.1 is a key hematopoietic cell transcription factor that can bind to the promoter region or enhancer region of the Dectin-1 gene and activate the transcription of the Dectin-1 gene. Dectin-1 is a C-type lectin receptor that mainly recognizes the cell wall component β-glucan of fungi. When Dectin-1 recognizes and binds to β-glucan on the surface of the pathogen, it will trigger its intracellular ITAM (immunoreceptor tyrosine activation motif).^27,28^ β3-integrin is an integrin receptor that participates in cell adhesion and signal transduction. Studies have found that Dectin-1 can form a complex with β3-integrin. This binding enhances the ability of cells to recognize and respond to pathogens. When Dectin-1 recognizes the pathogen, its ITAM motif will be phosphorylated by tyrosine kinases such as Lyn. Phosphorylated ITAM can provide a binding site for SYK (Spleen Tyrosine Kinase). At the same time, the signal transduction of β3-integrin enhances the activation effect of SYK. Therefore, the combined action of Dectin-1 and β3-integrin greatly enhances the activation of SYK in cells. After SYK is activated, through a series of signal cascade reactions, the NADPH oxidase complex is activated. The activation of this complex leads to the production of a large amount of ROS in the cell, thereby increasing oxidative stress.^29,30^

In this study, we found that the expression of PU.1, SYK, Dectin-1, β3-integrin, P22, P47, and P53 was significantly increased in C33A cells treated with 125I particle radiotherapy after overexpression of HSF1. Conversely, after inhibiting HSF1, the expression of PU.1, SYK, Dectin-1, β3-integrin, P22, P47, and P53 was significantly decreased. This confirmed that HSF1 is very important in the process of 125I particle radiotherapy-induced oxidative stress.

And ROS can promote the activity of deubiquitinating enzyme USP7 (Ubiquitin-Specific Protease 7) by oxidative modification or affecting cell signaling pathways. USP7 is an important deubiquitinating enzyme that can prevent proteins from being degraded by the 26S proteasome by removing ubiquitin chains on proteins. USP7 stabilizes the p53 protein by deubiquitination. p53 is a key tumor suppressor gene that has the functions of regulating the cell cycle, promoting apoptosis, and maintaining genomic stability. Under normal circumstances, the level of p53 is strictly regulated by MDM2 (an E3 ubiquitin ligase), which marks p53 with ubiquitin for degradation by the proteasome. USP7 prevents p53 from being degraded by removing the ubiquitin chain on it, thereby increasing the stability and activity of p53. Stable p53 transcription activates a series of downstream genes, and the proteins encoded by these genes are involved in processes such as cell cycle arrest, DNA repair, and apoptosis. Specifically, p53 can induce the expression of pro-apoptotic genes such as BAX, PUMA, and NOXA, while inhibiting the expression of anti-apoptotic genes such as Bcl-2, ultimately leading the cells to apoptosis.^31,32^

In the data set GSE56363, 334 significantly upregulated genes and 178 significantly downregulated genes were identified. Venn diagram analysis revealed 46 overlapping genes, among which 39 were significantly upregulated, suggesting that chemotherapy induces oxidative stress. GO and KEGG analyses conducted by ClusterProfiler indicated that these differentially expressed genes were significantly enriched in multiple biological pathways. GO analysis demonstrated that the differentially expressed genes were enriched in hemoglobin complexes, DNA repair complexes, and membrane rafts in terms of cellular components; in antioxidant activity, glutathione transferase activity, and oxygen binding in terms of molecular functions; and in response to oxidative stress, epithelial cell proliferation, and hydrogen peroxide metabolic processes in terms of biological processes. KEGG pathway analysis revealed that the differentially expressed genes were significantly enriched in the PI3K-Akt signaling pathway, chemical carcinogenesis-DNA adducts, and MAPK signaling pathway, among others. Correlation analysis revealed that HSF1 is lowly expressed in cervical cancer, and ROS can promote the expression of HSF1, which in turn promotes the expression of PU.1 (SPI1), and PU.1 (SPI1) promotes the expression of Dectin1 (CLEC7A). Additionally, ROS promotes the expression of USP7, which promotes the expression of P53, and ROS also promotes the expression of BRD4.Based on the results of Western blot, flow cytometry, CCK-8 assay, clone formation assay, and subcutaneous xenograft tumor experiment in nude mice, we can conclude that 125I particle radiotherapy enhances the expression of ROS, USP7, and P53 by promoting the HSF1/PU.1/SYK signaling pathway, thereby promoting the apoptosis of C33A cells and inhibiting their proliferation. Specifically, 125I particle radiotherapy significantly increased the relative protein expression levels of HSF1, PU.1, SYK, p-SYK, P47, gp91, p-USP7, and P53, and significantly increased the apoptosis rate of C33A cells, and decreased cell viability and proliferation ability. When HSF1 was specifically inhibited, these effects were significantly weakened, but when PU.1 was overexpressed simultaneously, these effects were significantly enhanced. Further, after the addition of the SYK-specific inhibitor R406, the expression levels of p-SYK, P47, gp91, p-USP7, and P53 and the apoptosis rate of cells were significantly decreased, and the cell viability and proliferation ability were significantly restored. The subcutaneous xenograft tumor experiment in nude mice verified these in vitro results, showing that 125I particle radiotherapy significantly inhibited the growth of tumors through the above signaling pathways. Combining these data, 125I particle radiotherapy effectively enhanced the expression of ROS, USP7, and P53 through the HSF1/PU.1/SYK signaling pathway, and inhibited the growth and proliferation of cervical cancer C33A cells, suggesting that the HSF1/PU.1/SYK signaling pathway may be the key mechanism of action of 125I particle radiotherapy.

In the management of recurrent cervical cancer, the combination of platinum-based chemotherapeutics, such as carboplatin and cisplatin, with paclitaxel continues to be the preferred first-line regimen, particularly for chemotherapy-naive patients with distant metastasis. This platinum-based chemotherapy protocol has exhibited a relatively high objective response rate (15%–46%). Nevertheless, non-platinum agents, including 5-fluorouracil, docetaxel, doxorubicin, gemcitabine, and mitomycin, as well as paclitaxel, irinotecan, topotecan, vincristine, and ifosfamide, have been assessed in several phase II trials, but generally demonstrate lower response rates and increased toxicity. Consequently, platinum-based chemotherapy remains the standard therapeutic approach.

Anti-angiogenic therapy has garnered considerable attention in recent years as a critical strategy. Bevacizumab, a multi-targeted VEGF tyrosine kinase inhibitor, has been extensively utilized in the treatment of recurrent cervical cancer and has shown excellent efficacy in terms of objective response rate (ORR). However, clinical trials have also evaluated other anti-angiogenic agents, such as Sunitinib, which have indicated limited monotherapy efficacy and a higher incidence of fistula formation, suggesting that monotherapy with anti-angiogenic agents may not be the optimal approach. Current ongoing research includes the combination of Apatinib with a platinum-based doublet, Nintedanib in conjunction with carboplatin and paclitaxel, and Nimotuzumab with concurrent chemoradiotherapy. Further investigation into these combination treatment strategies holds the potential to provide additional therapeutic options for patients with recurrent cervical cancer.

Immunotherapy, especially immune checkpoint inhibitors targeting PD-1 and PD-L1, has demonstrated substantial efficacy. Cemiplimab, an IgG1 monoclonal antibody against PD-1, has achieved an ORR of 44% in PD-L1 positive patients and an overall ORR of 33%. Moreover, a phase I/II clinical trial is currently underway to evaluate the combination of Cemiplimab and SNS-101 (an anti-VISTA antibody) in locally advanced, unresectable, or metastatic solid tumors, including recurrent cervical cancer. The outcomes of these trials will further substantiate the potential of immunotherapy in recurrent cervical cancer.^33^

Future research should concentrate on optimizing personalized treatment by considering the specific molecular alterations, comorbidities, and individual needs of patients to ensure a high quality of life. Given the elevated incidence and mortality rates of cervical cancer in developing countries, research should also emphasize the evaluation of treatment methods in these regions, assessing both feasibility and accessibility. Additionally, the combination of immunotherapy and anti-angiogenic therapy may offer enhanced efficacy for certain patient subgroups and warrants further exploration. While platinum-based chemotherapy remains the first-line treatment for recurrent cervical cancer, emerging anti-angiogenic and immunotherapy strategies exhibit significant potential. Upcoming clinical trials will further validate the efficacy and safety of these novel therapies, expanding the treatment landscape for cervical cancer patients.

The strength of this study lies in its comprehensive examination of the cytotoxic effects of 125I seed radiotherapy on cervical cancer cells, particularly through the upregulation of the HSF1/PU.1/SYK signaling pathway to enhance ROS/USP7/p53-mediated apoptosis. This research provides a novel theoretical foundation and practical guidelines for cervical cancer radiotherapy. Employing a range of experimental methods, including bioinformatics analysis, flow cytometry, CCK-8 assays, and colony formation assays, the study validates the effects of 125I seed radiotherapy from multiple dimensions, offering robust data support.

The results demonstrate that 125I seed radiotherapy significantly inhibits the invasion and migration of cervical cancer cells by upregulating the HSF1/PU.1/SYK signaling pathway. It also potentiates apoptosis and curtails cell proliferation. By upregulating the HSF1/PU.1/SYK signaling pathway, 125I seeds not only restrain the growth and proliferation of cervical cancer cells but also markedly diminish tumor invasion and migration. These findings suggest that 125I seeds may represent a more efficacious method of radiotherapy for cervical cancer.

Understanding the precise mechanisms of 125I seed radiotherapy can facilitate the development of personalized treatment plans tailored to individual patient characteristics. For instance, patients can be assessed for the activity of the HSF1/PU.1/SYK signaling pathway to determine their suitability for 125I seed treatment. This study not only enriches the theoretical research on cervical cancer radiotherapy mechanisms but also introduces new methodologies and approaches to improve treatment outcomes and quality of life for cervical cancer patients in clinical practice.

Future research can further corroborate these findings and explore broader clinical applications. However, the study also has certain limitations. While it initially investigates how 125I seed radiotherapy upregulates the HSF1/PU.1/SYK signaling pathway to enhance ROS/USP7/p53-mediated apoptosis, the exact mechanisms require more in-depth research, such as protein-level validation and the interactions between signaling pathways.

To sum up, our results show that 125I particles inhibit the progression of cervical cancer by up-regulating the HSF1/PU.1/SYK signaling pathway and enhancing the ROS/USP7/P53-mediated apoptotic response. These findings are expected to open up new future prospects and research directions for radiotherapy strategies for cervical cancer patients.

## Supplementary Information

Supplementary Information 1.

Supplementary Information 2.

Supplementary Information 3.

## Data Availability

The datasets extracted and/or analysed during the current study are available in the GSE56363 repository,GPL4133 Agilent-014850 Whole Human Genome Microarray 4 × 44 K G4112F (Feature Number version).
