# Supplementary figures and images for: 125I inhibits the progression of cervical cancer by upregulating the HSF1/PU.1/SYK signaling pathway and consequently enhancing the apoptotic response mediated by ROS/USP7/P53
Source: Sci Rep. 2025 May 21;15:17690. doi: 10.1038/s41598-025-99214-2 (PMC12095467; doi:10.1038/s41598-025-99214-2)

Fig 3A Supplementary figures

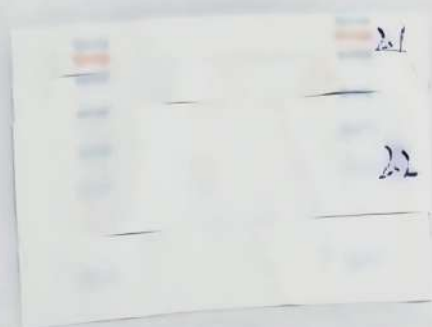

HSF1  
pectin-1

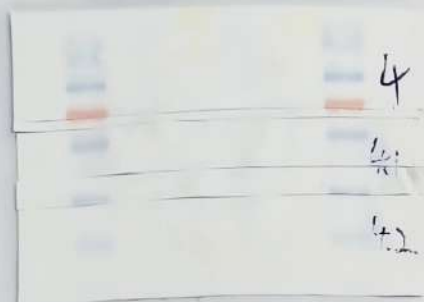

PU.1  
p47

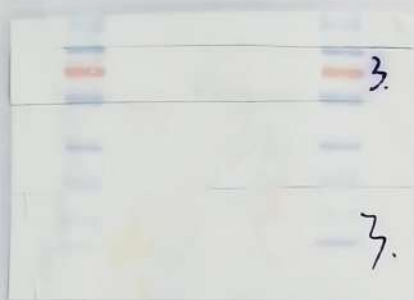

SYK

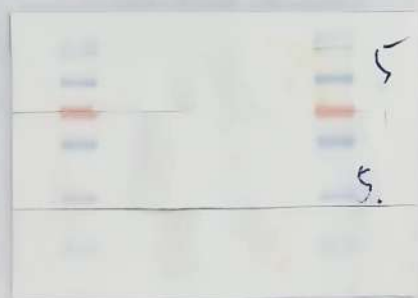

p13

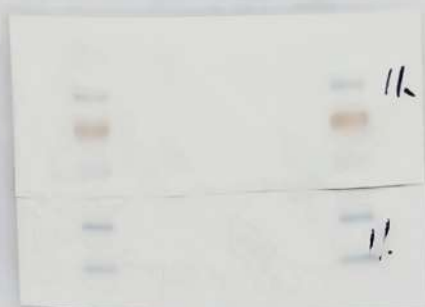

GAPDH

Fig 4A

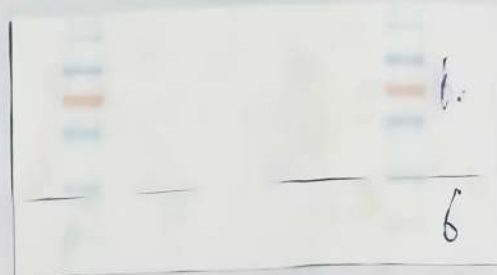

HSF1

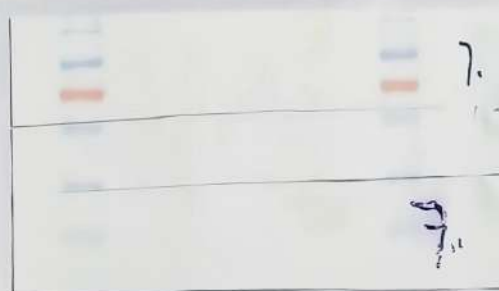

PU.1

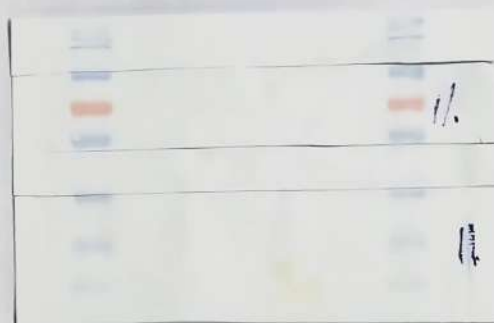

SYK

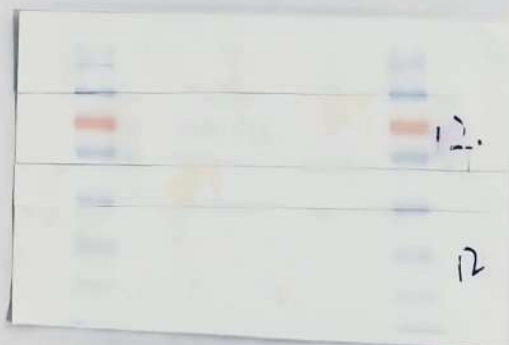

p-SYK

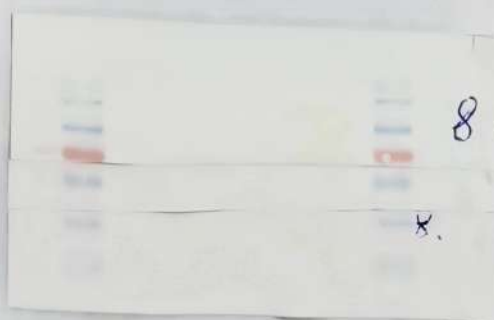

p47

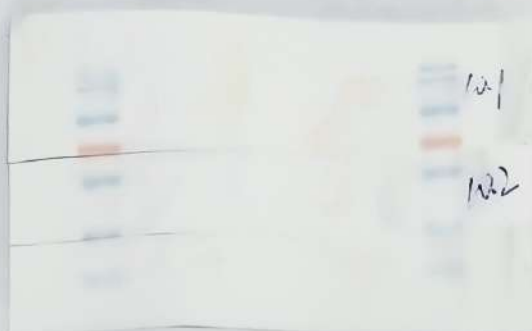

gp91

P-usb7

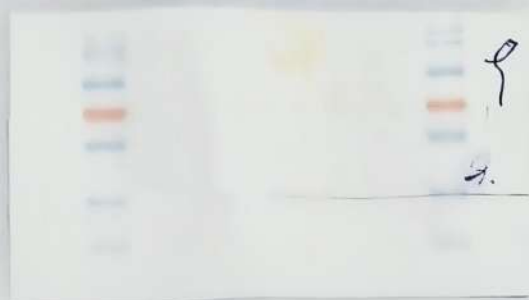

p53

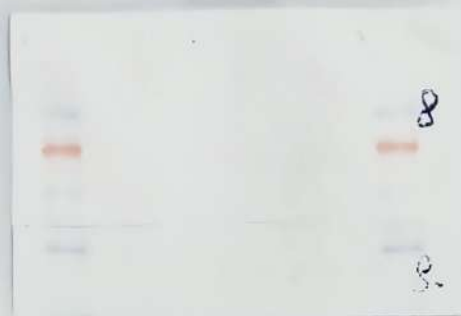

GAPDH

Supplement: Supplementary file 1 — Supplementary Information 1. [file 41598_2025_99214_MOESM1_ESM.pdf]
