# Supplementary material for: 125I inhibits the progression of cervical cancer by upregulating the HSF1/PU.1/SYK signaling pathway and consequently enhancing the apoptotic response mediated by ROS/USP7/P53
Source: Sci Rep. 2025 May 21;15:17690. doi: 10.1038/s41598-025-99214-2 (PMC12095467; doi:10.1038/s41598-025-99214-2)
Supplement: Supplementary file 2 — Supplementary Information 2. [file 41598_2025_99214_MOESM2_ESM.pdf]

Supplementary table 1: STR identification table for C33A cell

Cytogenetic Quality Assay

Cell Line Authentication Service

One、The cell name： C33A

Two、Methods: DNA was extracted using Axygen's genome extraction kit, amplified using the 21-STR amplification protocol, and tested for STR loci and the sex gene Amelogenin on an ABI 3730XL Genetic Analyzer.

Three、Sample genotyping results:

Genotyping results of STR loci and Amelogenin loci in cells						
Loci	STR information for sent cells			Cell Bank Cell STR Information		
	Name of Cells Sent for Examination： C33A			Cell Bank Cell Name： C33A		
	Allele1	Allele2	Allele3	Allele1	Allele2	Allele3
D5S818	11	12		11	12	
D13S317	13	13		13	13	
D7S820	10	10		10	10	
D16S539	13	14		13	14	
VWA	18	20		18	20	
TH01	7	8		7	8	
AMEL	X	X		X	X	
TPOX	9	9		9	9	
CSF1PO	12	12		12	12	
D12S391	18	27				
FGA	21	26				
D2S1338	23	25				
D21S11	29	31				
D18S51	15	18				
D8S1179	10	14				
D3S1358	16	16				
D6S1043	9	11	12.0			
PENTAE	6	8				

D19S433	11	13				
PENTAD	10	10				
D1S1656	15	16	17.0			

Note: The above figure shows the results of the comparison of the six genotyping loci of the assayed cells and cell banks.

Four、Conclusion: The DNA typing of this cell line was found to be an exact match in the cell line search, and the DSMZ database showed the cell name to be C33A. The DSMZ database shows the cell name as **C33A**. **No multiple alleles were found** in this cell line in this assay.
