# Supplementary material for: 125I inhibits the progression of cervical cancer by upregulating the HSF1/PU.1/SYK signaling pathway and consequently enhancing the apoptotic response mediated by ROS/USP7/P53
Source: Sci Rep. 2025 May 21;15:17690. doi: 10.1038/s41598-025-99214-2 (PMC12095467; doi:10.1038/s41598-025-99214-2)
Supplement: Supplementary file 3 — Supplementary Information 3. [file 41598_2025_99214_MOESM3_ESM.pdf]

Supplementary table 2: STR identification table for HELA cell

# Cytogenetic Quality Assay

## Cell Line Authentication Service

One、Cell name： HELA

Two、Methods: DNA was extracted using Axygen's genome extraction kit, amplified using the 21-STR amplification protocol, and tested for STR loci and the sex gene Amelogenin on an ABI 3730XL Genetic Analyzer.

Three、Sample genotyping results:

| Genotyping results of STR loci and Amelogenin loci in cells | | | | | | |
|---|---|---|---|---|---|---|
| Loci | STR information for sent cells | | | Cell Bank Cell STR Information | | |
| | Name of Cells Sent for Examination： HELA | | | Cell Bank Cell Name： HELA | | |
| | Allele1 | Allele2 | Allele3 | Allele1 | Allele2 | Allele3 |
| D5S818 | 11 | 12 | | 11 | 12 | |
| D13S317 | 12 | 13.3 | | 12 | 13.3 | |
| D7S820 | 8 | 12 | | 8 | 12 | |
| D16S539 | 9 | 10 | | 9 | 10 | |
| VWA | 16 | 18 | | 16 | 18 | |
| TH01 | 7 | 7 | | 7 | 7 | |
| AMEL | X | X | | X | X | |
| TPOX | 8 | 12 | | 8 | 12 | |
| CSF1PO | 9 | 10 | | 9 | 10 | |
| D12S391 | 20 | 24 | 25.0 | | | |
| FGA | 21 | 21 | | | | |
| D2S1338 | 17 | 17 | | | | |
| D21S11 | 27 | 28 | | | | |
| D18S51 | 16 | 16 | | | | |
| D8S1179 | 12 | 13 | | | | |
| D3S1358 | 15 | 18 | | | | |
| D6S1043 | 18 | 19 | | | | |
| PENTAE | 7 | 17 | | | | |

| | | | | | | |
|---|---|---|---|---|---|---|
| D19S433 | 13 | 14 | | | | |
| PENTAD | 8 | 15 | | | | |
| D1S1656 | 12 | 15 | | | | |

Note: The above figure shows the results of the comparison of the six genotyping loci of the assayed cells and cell banks.

Four、Conclusion: DNA typing of this cell line resulted in an exact match in the cell line search, and the DSMZ database showed the cell name to be **HELA**.This assay did **not find multiple alleles in this cell line**
